# A Practical Treatise on the Medical and Surgical Uses of Electricity

**Published:** 1888-05

**Authors:** 


### Book Reviews.

---

**A Practical Treatise on the Medical and Surgical Uses of Electricity,** including Localized and General Faradization, Localized and Central Faradization, Franklinization, Electrolysis and Galvano-Cautery. By Geo. M. Beard, A. M., M. D., and A. D. Rockwell, A. M., M. D. Sixth edition; revised by A. D. Rockwell, M. D., with nearly 200 illustrations. New York: Wm. Wood & Co., 56 and 58 Lafayette place. 1888. Matteson, Buffalo.

Since 1871, this work has stood pre-eminent in its particular field, and the progress of our knowledge has been recorded in the pages of its successive editions.

While electricity is far from being a panacea, yet its increasing range of usefulness in medicine may be best illustrated by reference to these various additions. In the second edition, the chapters on Electro-Physics and Physiology were largely re-written; the method of central galvanization described and illustrated, electro-surgery more fully treated, and the relation of electricity to the diseases of children and of the skin, considered in detail. In the third edition were given the highly-satisfactory results following the treatment of exophthalmic goitre by galvanization of the sympathetic, and of some of the sequelæ of acute diseases by general faradization. The chapter on Electro-Diagnosis also was largely rewritten. A fourth edition was rendered necessary by a revival of the use of Franklinic electricity, due to vastly improved appliances, and contained also the extraordinary results following the application of dynamic electricity to cases of extra-uterine pregnancy.

The fifth edition discussed facts hitherto, and even now, but little appreciated, concerning the induction coil, its varieties, and the differential indications for their use. These statements the author deems worthy of careful consideration, and believes that further experience will result in still more important as well as more definite deductions. Within the past two or three years, Apostoli, of Paris, has, by his experiments and the results that he has succeeded in obtaining, greatly enlarged the domain of electricity in gynecology. The revision in the present edition, therefore, has been mainly restricted to this subject, and the chapter on the Diseases of Women almost entirely recast. The methods through which these better results in gynecology are obtained, do but confirm the truth of the observation made in the preface to the third edition, to the effect that the real scientific basis for the use of electricity in medicine and surgery is found in electro-physics more than in electro-physiology.

